# Functional fantasies: the regulatory role of grandiose fantasizing in pathological narcissism

**DOI:** 10.3389/fpsyt.2023.1274545

**Published:** 2023-10-18

**Authors:** Ellen F. Finch, Jill M. Hooley

**Affiliations:** Department of Psychology, Harvard University, Cambridge, MA, United States

**Keywords:** narcissism, grandiose fantasy, affect regulation, self-esteem regulation, future thinking

## Abstract

**Introduction:**

Pathological narcissism is characterized by maladaptive efforts to maintain a bolstered but fragile sense of self. Clinical theory suggests that grandiose fantasizing may be one form of this self-regulation. However, no empirical research has directly assessed the regulatory function of grandiose fantasizing in narcissism. Here, we examine (1) whether people scoring higher in narcissism choose to engage in grandiose fantasizing to regulate themselves when they are feeling down and (2) whether grandiose fantasizing is a more efficacious self-esteem and affect regulator for people scoring higher in narcissism than it is for those scoring lower in narcissism.

**Methods:**

Adult participants (*N* = 189) completed a self-report measure of narcissism and were randomized to either a negative mood induction or filler task condition. Then, participants wrote about a future event to make themselves feel better, choosing between a positive affect word or a grandiose word to guide their writing. Throughout the study, participants reported their state positive and negative affect and self-esteem. A secondary sample (*N* = 128) of adult participants rated the future event writing of the original participants.

**Results:**

Supporting the validity of the study design, grandiose future events significantly differed from positive future events (e.g., they were rated by independent raters as less plausible, more ambitious, more active, and occurring further in the future). Participants scoring higher in narcissism and participants who experienced larger increases in negative affect were more likely to choose to engage in grandiose fantasizing. Grandiose fantasizing was more effective at decreasing negative affect among participants scoring higher in narcissism than those scoring lower in narcissism, whereas positive future thinking was equally effective at decreasing negative affect across levels of narcissism.

**Discussion:**

This study demonstrates that people scoring higher in narcissism are more likely to choose to engage in grandiose fantasizing to make themselves feel better. It further demonstrates that grandiose fantasizing is a more efficacious affect regulator for those scoring higher in narcissism.

## 1. Introduction

Pathological narcissism is characterized by maladaptive efforts to maintain a bolstered but fragile sense of self (1, 2). This self-regulation occurs both inter-and intra-personally. Interpersonal regulation in pathological narcissism has been widely studied, with research demonstrating that people scoring high in pathological narcissism seek validation from their social environment, such as surrounding themselves with people who will admire them and acting with aggression and anger towards others when insulted [for summary see (1)]. Less studied, however, is intrapersonal regulation, or how people scoring high in narcissism internally regulate their enhanced sense of self. A longstanding hypothesis is that grandiose fantasizing – defined as engaging in fantasies of success, power, brilliance, or beauty (3) – is a common and critical form of intrapersonal self-regulation among people with pathological narcissism.

Grandiose fantasizing has been considered a core feature of narcissism since the early writing of Freud (4), who believed that narcissism was characterized by frequent generation of grandiose images of the self. Subsequent theoretical work, based on numerous case studies, suggests that patients with Narcissistic Personality Disorder (NPD) often retreat into fantasy worlds in which they are admired, respected, and dominant [e.g., (5, 6)]. The centrality of grandiose fantasizing to clinical conceptualizations of narcissism is so strong that engaging in grandiose fantasizing was included as a diagnostic criterion of NPD when the disorder was added to the DSM-III in 1980 (7). It remains in the DSM to this day.

Empirical work has confirmed that grandiose fantasizing is fundamental to NPD and narcissism more broadly. Grandiose fantasizing is among the most common NPD criteria, present in approximately 79% of those diagnosed with NPD (8). Multiple factor analyses [e.g., (9–11)] and network analyses (12, 13) suggest that grandiose fantasizing lies at the core of narcissism. Further, these studies suggest that grandiose fantasizing is tightly linked to both major dimensions of the bifurcated model of narcissism: vulnerability (e.g., feelings of inferiority, fear of failing and losing control) and grandiosity (e.g., arrogance, entitlement) [for discussion of these two dimensions, see (1, 14, 15)]. Both grandiose and vulnerable narcissism are similarly associated with the related construct of a fantasy defense mechanism characterized by indulging in excessive daydreaming as a substitute for active problem solving (1, 14–16). Above and beyond many other NPD criteria, grandiose fantasizing is highly associated with negative psychological correlates of NPD, including depression, anxiety, stress, neuroticism, shame, identity diffusion, and aggression (11, 17), and negatively correlated with self-esteem (13, 17). These psychometric analyses are complemented by experimental research finding that the mind wandering content of those high in narcissism tends to be future oriented, self-revelatory, positive, and achievement focused (18, 19), which may reflect grandiose fantasizing.

Together, this work highlights how closely linked grandiose fantasizing is to narcissism. Little research has examined *when* and *why* grandiose fantasizing occurs, however. In other words, under what circumstances do people scoring high in narcissism engage in grandiose fantasizing, and what function does it serve? Clinical theory posits that people scoring high in narcissism use grandiose fantasizing as a self-regulation tool following a narcissistic injury (18). Theoretical support for this idea comes from numerous psychoanalytic thinkers [e.g., (5, 20, 21)]. Although, like Freud, they understood grandiose fantasy as a representation of the grandiose self, they also conceptualized grandiose fantasy as a functional cognitive tool to regulate self-esteem. Fantasizing about future successes can reinstate a sense of grandiosity after an inflated ego is wounded. For example, if a person scoring high in narcissism feels insulted in the moment, they may fantasize about being widely admired to help themselves feel better. Accordingly, grandiose fantasy’s regulatory role is often framed as a defense mechanism (16, 18) or character defense (20), particularly in psychoanalytically informed literature.

There is limited empirical work supporting the idea that grandiose fantasy serves a regulatory function, however. No research has directly assessed if grandiose fantasizing effectively regulates a narcissistic sense of self or any related constructs (e.g., self-esteem; identity; affect). Most closely, Raskin and Novacek (18) found that, among participants scoring high in narcissism, grandiose fantasizing was associated with daily stress, indicating a possible compensatory relationship between stress level and grandiose fantasizing. Relatedly, Baumeister et al. (22) completed a series of studies showing that, following an ego-threat, people scoring high in self-esteem (a concept related to, but distinct from, narcissism) tended to set risky goals for themselves that were beyond their performance capabilities. This again supports the idea that overly ambitious future thinking, which has similarities to grandiose fantasizing, may be compensatory. More recently, structural equation modeling (23) demonstrated that characterological shame mediates the relationship between vulnerable narcissism and maladaptive daydreaming, defined as “extensive fantasy activity that replaces human interaction and/or interferes with…functioning” (24, p. 199). This cross-sectional mediation relationship may mean that individuals high in vulnerable narcissism engage in maladaptive daydreaming to protect against feelings of shame, again suggesting a regulatory function. Each of these studies lends correlational support to the idea that grandiose fantasy may serve in a regulatory capacity following a narcissistic injury.

To date, however, no research has demonstrated this in an experimental, or causal, context. This may be due to challenges inherent in studying grandiose fantasy experimentally. Although the DSM-5 offers a definition that facilitates clinical diagnosis [i.e., “is preoccupied with fantasies of unlimited success, power, brilliance, beauty, or ideal love” (3)], grandiose fantasies have yet to be well operationalized. In other words, there is no established way to determine if a person’s future hopes can be characterized as grandiose fantasy. Fantasy also tends to be specific to the individual, and thus must be self-generated. For example, whereas one individual may have a recurring fantasy about being a professional athlete, another person may have no interest in an athletic career. Thus, it is difficult to experimentally induce grandiose fantasizing across multiple participants.

Measurement presents a further challenge. Many self-report narcissism measures include items evaluating grandiose fantasies [e.g., “I daydream about someday becoming famous” in the Five-Factor Narcissism Inventory (25) and “I often fantasize about being admired and respected” in the Pathological Narcissism Inventory (11)]. In addition, there are several self-report scales that assess related constructs, such as maladaptive daydreaming (24, 26), fantasy styles (27), or pursuit of unrealistic goals (28). However, each of these measures assess general tendencies for fantasy. To establish a regulatory function, it is necessary to identify the use of grandiose fantasizing in a given moment. Yet there is currently no method to induce or evaluate momentary grandiose fantasizing.

In the present study, we explore grandiose fantasizing as it relates to narcissism using a novel choice paradigm that addresses these methodological challenges. Participants across a wide range of narcissism were recruited. Following randomization to a negative affect and self-esteem induction or filler task, participants were instructed to regulate themselves by choosing to engage in *either* grandiose fantasizing or positive future thinking. Through employing positive future thinking as a comparator condition, we can ensure that any findings are unique to grandiose fantasizing and not positive future thinking more broadly. Following participants’ choices, they engaged in their chosen regulation technique through writing about the grandiose or positive future event. Participants reported state self-esteem and affect throughout the study.

This experimental framework allows us to answer two primary research questions. First, do people scoring higher in narcissism choose to engage in grandiose fantasizing to regulate themselves when they are feeling down? Although correlational evidence has established that people scoring higher in narcissism are more likely to engage in grandiose fantasizing in general and also when stressed [e.g., (18)], we do not yet have experimental evidence that this is a regulation tool utilized in moments of lowered self esteem or worsening affect. We predicted that people scoring higher in narcissism would be more likely to choose grandiose fantasizing as a regulation tool than those scoring lower in narcissism. Further, if grandiose fantasizing is a regulation tool and not just a general tendency, participants scoring higher in narcissism should be more likely to pursue grandiose fantasizing when feeling down. Thus, we predicted that this effect would be stronger following a larger decrease in self-esteem and/or worsening of affect.

The second question we addressed is whether grandiose fantasizing is a more efficacious self-esteem and affect regulator for people scoring higher in narcissism. Given that grandiose fantasizing is a well-established behavior among people scoring higher in narcissism, it is likely reinforced through improving affect and/or self-esteem, at least in the short term. No research, however, has examined how the short-term effects of engaging in grandiose fantasizing vary with level of narcissism. We predicted that grandiose fantasizing would improve short-term affect and self-esteem more so than positive future thinking, particularly for people scoring higher in narcissism.

These primary research questions and hypotheses relate to overall pathological narcissism, a multidimensional construct that includes both grandiose and vulnerable features. Given that grandiosity and vulnerability often show divergent patterns [for review see (29, 30)], we also consider vulnerable and grandiose narcissism individually in exploratory analyses. This approach allows us to determine if results are driven by a single narcissism dimension rather than the overall construct.

Lastly, our study also sought to better characterize how grandiose fantasizing might differ from positive future thinking, and thus begin laying the groundwork for formal operationalization. We used independent raters to evaluate the grandiose and positive future writing on several dimensions chosen for their face validity (e.g., ambitiousness, agency, plausibility). In addition to exploring possible metrics with which to operationalize grandiose fantasizing, these external ratings also allowed us to test if grandiose future events and positive future events were qualitatively different, thereby supporting the validity of our choice task.

Though grandiose fantasizing is a core feature of narcissism, little empirical work has examined why this is. This experimental study tests the longstanding clinical theory of the regulatory function of grandiose fantasizing in narcissism. This study also explores potential features of grandiose fantasizing, laying the groundwork for future research in this area.

## 2. Materials and methods

### 2.1. Participants

#### 2.1.1. Primary sample

One hundred and ninety-three healthy adults were recruited from Prolific (31). To meet inclusion criteria, participants were required to: be between the ages of 18–40 years, be fluent in English, have no long-term health conditions or disabilities, currently reside in the United States, and have a historical Prolific approval rating of above 90%, as determined by their Prolific profiles. Those meeting these inclusion criteria could elect to participate after reading a study description. The study description informed participants that they would be completing an online study in which they would be asked to answer questions about themselves and their mood, as well as write about their life.

Following data collection, participant data were removed if the participant responded randomly or illogically, failed more than one of four attention checks that were scattered throughout the questionnaires, or had a history of traumatic brain injury. One participant was removed for random/illogical responding (i.e., writing did not respond to prompts; contradictory responding in questionnaires), one participant was removed for failing more than one attention check, and two participants were removed for reporting a history of traumatic brain injury. This left a final *N* of 189. Given the limited prior work in this domain, sample size was determined using existing guidelines for logistic regression analyses (32), which recommend 50 occurrences in each outcome category. Therefore, data were collected until each regulation technique (i.e., grandiose fantasizing and positive future thinking) had been chosen by at least 50 participants.

Participants’ average age was 29.48 years (SD = 5.73). Approximately one half of participants (49.74%) identified as cisgender female, 47.09% identified as cisgender male, 1.59% (three participants) identified as transgender female, 1.06% (two participants) as multiple gender identities, and one participant as non-binary/genderqueer. Most participants were heterosexual (79.90%), followed by bisexual (11.64%), gay/lesbian/homosexual (3.70%), pansexual (3.17%), or unsure (1.59%). Regarding race and ethnicity, two thirds of the sample (66.66%) identified as White, 8.47% identified as Black or African American, 8.47% identified as multiple races/ethnicities, 7.41% identified as Asian, 5.82% identified as Hispanic, Latino, or Spanish, 1.58% identified as Middle Eastern or North African, and 1.06% identified as American Indian or Alaskan Native. One half (50.26%) of participants were employed full time, another 13.76% were employed part time, and 16.93% were unemployed; 8.99% of participants were students, 6.35% were self-employed, and 3.70% were homemakers.

#### 2.1.2. Secondary sample

A second sample (*N* = 128) was recruited from Prolific using the same inclusion and exclusion criteria as the primary sample. Participants from the second sample served as independent external raters of the writing of the primary sample. Full demographic information for the secondary sample is available in Supplementary Table S1 of the Online Supplementary Materials.

### 2.2. Materials

#### 2.2.1. Five Factor Narcissism Inventory – Short Form (FFNI-SF)

The FFNI-SF (31) is a 60-item version of the original Five-Factor Narcissism Inventory [FFNI; (32)]. Participants rate the degree to which each statement captures them on a five-point Likert scale (1 = disagree strongly, 5 = agree strongly). Example statements include “It is easy to get people to do what I want” and “I am a superior person.” These 60 statements assess 15 distinct narcissism traits (e.g., authoritativeness, entitlement, reactive anger, need for admiration), which are then all summed to provide a total narcissism score. Higher scores indicate higher levels of pathological narcissism. Subsets of the 15 traits can be summed to provide grandiose narcissism and vulnerable narcissism scores. The FFNI-SF has been well validated across diverse samples and shows comparable reliability and validity to the original FFNI (33). Further, it has stronger incremental validity in predicting NPD traits than other pathological narcissism scales (25). In the present samples, the internal reliability of the FFNI-SF was excellent (Cronbach’s alpha = 0.915).

#### 2.2.2. Rosenberg Self Esteem Scale (RSES)

The RSES (34) is a 10-item, self-report measure of global self-worth (34). Participants rate statements of general feelings about themselves on a four-point Likert scale (1 = Strongly Agree, 4 = Strongly Disagree). Example items include “On the whole, I am satisfied with myself” and “I feel I do not have much to be proud of.” Following reverse scoring of five items, responses are summed to generate a total score with a higher score reflecting higher self-esteem. The RSES has been widely used and well-validated across various populations, languages, and settings [for reviews see (35, 36)] and had excellent internal reliability in the present sample (Cronbach’s alpha = 0.932).

#### 2.2.3. Depression and Anxiety Stress Scale-21 (DASS-21)

The DASS-21 (37) is a 21-item self-report measure derived from the DASS-42. It assesses depression, anxiety, and stress. Participants rate how much 21 statements applied to them over the past week. Example items include “I tended to over-react to situations” and “I felt downhearted and blue.” The DASS-21 is widely used and well-validated across numerous samples (38–40). In the present sample, the DASS-21 had excellent overall internal reliability (Cronbach’s alpha = 0.953), and strong reliability for each of its three subscales (anxiety subscale Cronbach’s alpha = 0.862; depression subscale Cronbach’s alpha = 0.947; and stress subscale Cronbach’s alpha = 0.891).

### 2.3. Procedure

All study procedures were approved by the Harvard University Institutional Review Board.

#### 2.3.1. Primary study

The study was completed online via Qualtrics (41). Participants first completed demographics measures and a battery of questionnaires, including the FFNI-SF, RSES, and DASS-21. Then, participants were randomized to complete either a negative mood induction or a filler task. Both a negative mood induction and neutral filler task were used so that there would be a wide range of changes in affect and self-esteem across participants, ranging from feeling much worse than at baseline to feeling relatively the same as at baseline. With this design, we could treat changes in affect and esteem as continuous variables. The negative mood induction has previously been used in our lab [e.g., (42, 43)] and was originally adapted from Bastian et al. (44). Participants randomized to the negative mood induction were instructed to first, “think about all the times in which you have failed or let yourself down,” then to identify the event that had had the most negative impact on them, and third, to write about that event and its consequences for three minutes. The filler task instructed participants to write down at least 15–20 objects in the room they were currently in for 3 min. The design of the filler task meant that participants in both the negative mood induction and filler task conditions were writing for 3 min.

Next, participants completed the future thinking choice and writing task. A novel choice task was developed to experimentally assess tendencies for grandiose fantasizing without priming participants. Participants were told they would respond to a writing prompt to make them feel good about themselves. Then, participants selected a word to complete their writing prompt: *“To make myself feel good, I am going to write about a time in the future when I will be: ______.”* The participant chose the word from a list of six adjectives. Three adjectives (attentive, inspired, enthusiastic) were positive affect words from the Positive and Negative Affect Scale [PANAS; (45)] and three adjectives (extraordinary, powerful, superior) were grandiose words from the Narcissistic Grandiosity Scale [NGS; (46)].^2^ Words were presented in a randomized order. The type (i.e., positive affect or grandiose) of the chosen word served as the main outcome variable of interest. After selecting a word, participants wrote for 3 min in response to their chosen prompt.

Thus, within this paradigm all participants wrote about a future event that they believed would make them feel good. Some chose to write about a future event when they would feel positive affect (e.g., *“I will be enthusiastic when I go on a vacation to Las Vegas. I will wander around the streets and eat delicious food…”*) whereas others chose to write about a future event when they would embody grandiose traits (e.g., “*I will have launched my company. I will be impacting millions of people and changing lives…”*). Future writing prompted by positive affect words will be referred to as positive affect events, and future writing prompted by grandiose words will be referred to as grandiose events.

State positive affect, state negative affect, and state self-esteem were assessed throughout the study using three Visual Analog Scales (VASs; 0 = “not at all positive/not at all negative/feel not at all good about myself”; 100 = “extremely positive/extremely negative/feel extremely good about myself”). VASs were completed (1) before beginning the questionnaire battery, (2) after the negative mood induction or filler task, and (3) after completing the future thinking choice and writing task. Altogether, the study took approximately 20 min and participants were paid $4.00 for their time.

#### 2.3.2. Secondary study

The secondary study was also completed via Qualtrics. After providing demographic information, participants were shown a random subset of 15 of the future events written in the primary study. All writing was deidentified. Participants rated the written future events on: (1) agency (i.e., *How active* vs. *passive is the event? In other words, is the writer making the event happen* (i.e.*, active*)*? Or is the event happening to the writer* (i.e.*, passive*)*?*), (2) ambitiousness (i.e., *How ambitious is this event? In other words, how bold or difficult to achieve is it?*), (3) distinctiveness (i.e., *How distinctive is this event? In other words, how unusual or unique is it?*), (4) emotional tone (i.e., *How overall negative or positive is the emotional tone of the event?*), (5) elaboration (i.e., *How well elaborated is this event? In other words, how well thought out, or detailed, is it?*), (6) plausibility for the average adult (i.e., *In the real world, how likely is it that this event will actually occur? In other words, how plausible, or viable, is this event for the average adult?*), (7) plausibility for the rater (i.e., *How likely is it that this event could occur in YOUR life, if you really wanted it to? In other words, how plausible, or viable, is this event for YOU?*), and (8) temporal proximity (i.e., *How far in the future is this event? In other words, how long until this event occurs?*). Each rating was completed on a 7-point Likert-type scale. On average, each future event was rated by 10 participants. Altogether, the secondary study took approximately 30 min and participants were paid $6.00 for their time.

### 2.4. Analytic approach

All analyses were conducted in R (RStudio version 2023.03.0). All plots were generated using the “plot_model” function in *sjPlot* package [version 2.8.12] (47). The FFNI-SF total score, FFNI-SF grandiose subscore, and FFNI-SF vulnerable subscore were each scaled for ease of interpretability and were treated as continuous variables. There were no missing data. Welch’s two-sample t-tests and chi-square analyses were used to evaluate any demographic differences between participants who chose a grandiose word and participants who chose a positive affect word. Any significant demographic differences between groups would be included as covariates in main analyses. Welch’s two-sample *t*-tests were used to assess group differences between positive affect events and grandiose events in secondary sample ratings (e.g., plausibility, proximity).

Robust binomial logistic regression [using the “glmrob” function in the *robustbase* package (version 0.95–0) (48)] was used to evaluate if grandiose fantasizing was chosen as a regulation tool by people scoring higher, but not lower, in narcissism when they were feeling down. Selected word type (positive affect word vs. grandiose word) in the future thinking choice paradigm was the outcome of interest and was numerically represented as either zero (positive affects words) or one (grandiose words). Main effects of narcissism (FFNI-SF total score) and change in affect and self-esteem immediately prior to word choice task (VAS change score for negative affect, VAS change score for positive affect, and VAS change score for self-esteem) were assessed. Then, interaction effects between narcissism and change in positive affect, change in negative affect, and change in self-esteem were assessed.

Robust linear regression [using the “rlm” function in the *MASS* package [version 7.2–58.2] (49)] was used to assess if engaging in grandiose fantasizing was an efficacious affect and self-esteem regulator among those scoring highest in narcissism. Changes from before to after the future thinking paradigm in positive affect, negative affect, and self-esteem were the outcomes of interest. First, main effects of narcissism and word choice type (positive affect word vs. grandiose word) were each tested. Second, interaction effects between narcissism and word choice type were tested.

To explore if the effects of narcissism are driven by either grandiose or vulnerable narcissism, any model with total narcissism as a predictor variable was repeated twice, with total narcissism replaced by grandiose narcissism and vulnerable narcissism.

For all statistical models, model assumptions of independent observations and multicollinearity were met. For binomial logistic regressions, the Box-Tidwell test showed that all models met the assumption of linear relationships between explanatory variables and logit of the response variable. To ensure that high leverage data points were not overly influential while also not unnecessarily removing participants, we used robust statistics for all models. Of note, the vast majority of reported significant effects remained significant (*p* < 0.05) when using non-robust modeling. Any effects that did not maintain significance with non-robust modeling are denoted with an asterisk (*) in results.

To confirm that findings were not driven by related clinical variables, all models that include narcissism as an independent variable were also tested with the addition of three covariates: self-esteem [as measured by total score of the Rosenberg Self Esteem Scale (34)], depression [as measured by the depression subscale of the Depression and Anxiety Stress Scale-21 (37)], and anxiety [as measured by the anxiety subscale of the Depression and Anxiety Stress Scale-21 (37)]. The inclusion of these covariates did not alter the significance of any reported findings, therefore the most parsimonious models with no additional covariates are reported here.

## 3. Results

### 3.1. Manipulation checks and group differences

Participants’ total narcissism scores (FFNI-SF total score, prior to being scaled) ranged from 80 to 220 (*M* = 150.84, SD = 30.25), reflecting a wide range of pathological narcissism. Fifty participants chose a grandiose word and 139 participants chose a positive affect word. The grandiose and positive affect groups did not significantly differ in age, sex, gender, race/ethnicity, sexuality, or employment status (all *p*s > 0.05). Therefore, demographic characteristics were not included as covariates in any models.

As predicted, Welch’s two-sample t-tests showed that in comparison to positive affect events, grandiose events were rated as less plausible for the average adult (*t* (73.23) = −3.24, *p* = 0.002), less plausible for the secondary rater (*t* (89.44) = −2.36, *p* = 0.020), more ambitious (*t* (105.01) = 5.70, *p* < 0.001), more active (vs. passive) (*t* (92.2) = 2.14, *p* = 0.035), occurring further in the future (*t* (84.38) = 3.78, *p* < 0.001), and having a more negative emotional tone (*t* (73.23) = −3.24, *p* = 0.002). There were no significant differences in event elaboration (*t* (90.19) = 0.90, *p* = 0.369) or distinctiveness (*t* (90.74) = 0.91, *p* = 0.364).

### 3.2. Negative mood induction and filler task

The negative mood induction and filler task groups were collapsed so that change in affect/esteem could be treated as a single, continuous variable. As intended, randomizing participants to either a negative mood induction or a filler task resulted in a wide range of changes in affect and self-esteem. From baseline to post negative mood induction or filler task, there was, on average, a significant increase in negative affect (range: −30 – 92, *M_change_* = 9.74, SD*_change_* = 21.87, *t* (188) = −6.12, *p* < 0.001), a significant decrease in positive affect (range: −92 – 33, *M_change_* = −8.50, SD*_change_* = 20.26, *t* (188) = 5.77, *p* < 0.001), and a significant decrease in state self-esteem (range: −81 – 43, *M* = −5.96*_change_*, SD*_change_* = 19.18, *t* (188) = 4.27, *p* < 0.001).

### 3.3. Future writing word choice (grandiose vs. positive affect)

**Figure 1:**
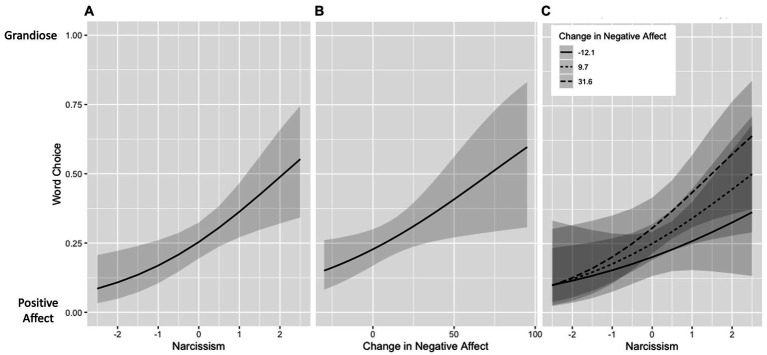
Predicted probability of word choice (grandiose vs. positive affect). Figure depicts the binomial logistic regressions evaluating word choice type (section 3.3). Higher values on the y-axis indicate a higher likelihood of choosing a grandiose word. Panel **(A)** depicts the relationship between narcissism and word choice. Panel **(B)** depicts the relationship between change in negative affect and word choice. Panel **(C)** depicts the relationship between the interaction of narcissism and change in negative affect and word choice (not a statistically significant interaction effect). “Narcissism” = scaled Five Factor Narcissism Inventory – Short Form total score; higher score indicates higher level of narcissism. “Change in Negative Affect” = change in negative affect (0–100 visual analog scale) from baseline to post-negative mood induction or filler task. Shaded area represents 95% confidence intervals.

As hypothesized, binomial logistic regression showed that narcissism predicted chosen word type [*OR* = 1.68, 95% CI (1.18, 2.38), *p* = 0.004]. People scoring higher in narcissism were more likely to choose a grandiose word than those scoring lower in narcissism (Figure 1A). The same held true for grandiose narcissism [*OR* = 1.63, 95% CI (1.15, 2.29), *p* = 0.006] but not for vulnerable narcissism [*OR* = 1.02, 95% CI (0.99, 1.04), *p* = 0.232]. Further, the magnitude of the increase in negative affect [*OR* = 1.02, 95% CI (1.00, 1.03), *p* = 0.018] (Figure 1B) and decrease in positive affect [*OR* = 0.98, 95% CI (0.97, 1.00), *p* = 0.043]* immediately prior to the choice task predicted chosen word type; larger increases in negative affect and larger decreases in positive affect were associated with an increased likelihood of choosing a grandiose word. Change in state self-esteem did not reach significance in predicting chosen word type [*OR* = 0.99, 95% CI (0.97, 1.00), *p* = 0.081].

Contrary to our hypotheses, word type was not significantly predicted by interactions between narcissism and change in negative affect [*OR* = 1.01, 95% CI (0.99, 1.02), *p* = 0.507] (Figure 1C), change in positive affect [*OR* = 1.00, 95% CI (0.99, 1.02), *p* = 0.669], or change in state self-esteem [*OR* = 1.00, 95% CI (0.98, 1.02), *p* = 0.999]. This was also the case for grandiose and vulnerable narcissism (all *p*s > 0.05).

### 3.4. Affect and esteem regulation

Robust linear regressions showed that following future writing, there were no main effects of narcissism on change in negative affect [*β* = −1.01, 95% CI (−2.85, 0.82), *p* = 0.278], positive affect [*β* = 1.55, 95% CI (−0.23, 3.34), *p* = 0.088], or self-esteem [*β* = 1.47, 95% CI(−0.39, 3.34), *p* = 0.121]. The same was true for grandiose and vulnerable narcissism (all *p*s > 0.05). There was a main effect of word type on change in negative affect [*β* = 5.84, 95% CI (1.65, 10.04), *p* = 0.007] and positive affect [*β* = −5.41, 95% CI (−9.43, −1.40), *p* = 0.009], such that people who wrote about grandiose events saw larger decreases in negative affect and larger increases in positive affect than those who wrote about positive affect events. Word choice type did not significantly predict changes in state self-esteem [*β* = −3.10, 95% CI (−7.21, −1.01), *p* = 0.139].

**Figure 2:**
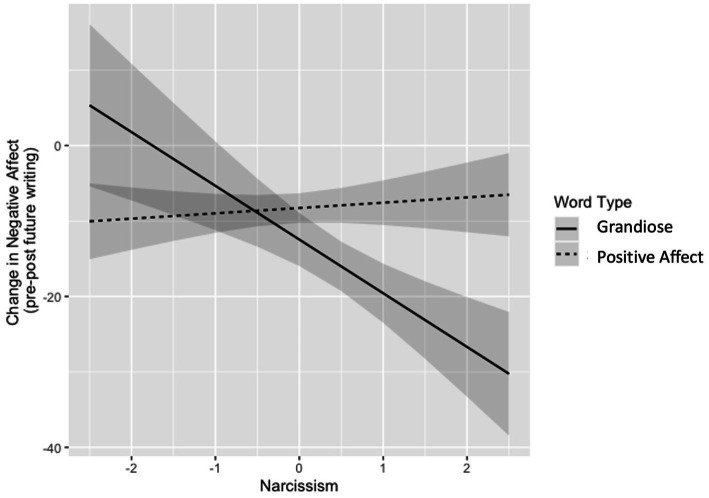
Interaction between narcissism and word type predicting change in negative affect. Figure depicts the predicted values of the linear model evaluating the interaction between narcissism and word type predicting change in negative affect (section 3.4). “Narcissism” (x-axis) = scaled Five Factor Narcissism Inventory – Short Form total score; higher score indicates higher level of narcissism. “Change in Negative Affect” (y-axis) = change in negative affect (0–100 visual analog scale) from pre to post future writing task; lower score indicates larger reductions in negative affect. Shaded area represents 95% confidence interval.

Aligning with our hypotheses, there was a significant interaction between narcissism and word choice type in predicting change in negative affect [*β* = 7.82, 95% CI (3.42 12.23), *p* = 0.001] (Figure 2). Simple slope analyses of this interaction effect show that writing about grandiose events was more effective at decreasing negative affect among people scoring higher in narcissism than those scoring lower in narcissism [*β* = −6.91, CI (−12.60, −1.22), *p* = 0.018], whereas writing about positive affect events was equally effective at decreasing negative affect across levels of narcissism [*β* = 0.69, CI (−1.23, 2.61), *p* = 0.480]. Further, change in negative affect was significantly predicted by the interaction between word choice type and grandiose narcissism [*β* = 4.90, 95% CI (0.50, 9.31), *p* = 0.029]* and word choice type and vulnerable narcissism [*β* = 4.47, 95% CI (0.60, 8.34), *p* = 0.024]*. There were no significant interactions for narcissism and word choice type on change in positive affect [*β* = −2.05, 95% CI (−6.38, 2.27), *p* = 0.350] or change in self-esteem [*β* = −0.50, 95% CI (−4.98, 3.99), *p* = 0.828]. This remained true for grandiose narcissism and vulnerable narcissism (all *p*s > 0.05).

## 4. Discussion

This study provides new insights into the function of grandiose fantasizing in pathological narcissism. People scoring higher in narcissism were more likely to *choose* to engage in grandiose fantasizing over generally positive future thinking when trying to make themselves feel better. This aligns with prior work establishing a strong general tendency for grandiose fantasizing among people scoring high in narcissism [e.g., (8, 11, 13)]. But, more specifically, we demonstrate that grandiose fantasizing is used as a conscious choice when people scoring high in narcissism try to feel better. We also found that grandiose fantasizing is a more efficacious negative affect regulator for people scoring higher in narcissism than it is for people scoring lower in narcissism. The same was not true for positive future events – change in negative affect following positive future thinking did not vary with narcissism. Together, this indicates that grandiose fantasizing has a specific role in narcissistic affect regulation. Overall, this supports the longstanding clinical theory that grandiose fantasy is used as an effective affect regulation tool among people high in narcissism.

Moreover, regardless of their level of narcissism, people are more likely to choose to engage in grandiose fantasizing (versus positive future thinking) following a worsening of affect – the larger the spike in negative affect, the more likely they are to choose grandiose fantasizing. This unexpected finding suggests that grandiose fantasizing may not only be an affect regulation technique for people scoring high in narcissism, but for anyone who is feeling down. This is supported by the psychoanalytic literature on defense mechanisms, which identifies fantasizing as a general defense mechanism that is not specific to narcissism [see (50)]. The lack of a significant interaction between narcissism and change in affect in predicting word choice contrasts our hypothesis that people scoring high in narcissism who also experienced a worsening of affect would be particularly likely to choose a grandiose word. However, the non-significant effect was in the hypothesized direction (see Figure 1C), suggesting that our sample size may not have been large enough to detect the effect.

Exploratory analyses demonstrated that overall narcissism had larger effect sizes than either grandiose or vulnerable individually across all models. This pattern suggests that people with a combination of grandiose and vulnerable traits – who are also typically those with more severe narcissism (14, 51) – are the most likely to engage in grandiose fantasizing. It also suggests that grandiose fantasizing is most effective for people with both these traits. This aligns with prior research reporting that grandiose fantasies are present in both grandiose and vulnerable narcissism (11, 13). Interestingly, this suggests that grandiose fantasizing stands in contrast to many other correlates of narcissism. Frequently, correlates that are present for grandiosity are absent for vulnerability, and vice versa [for summary see (29, 30)]. Grandiose fantasizing seems to be salient for both.

Prior work largely frames grandiose fantasizing as a regulator of self-esteem [e.g., (5, 18)] or self-image (20), with little mention of its potential to regulate related phenomena, such as affect. Yet, self-esteem and affect influence, or are at least closely associated with each other in narcissism [for review see (52, 53)]. Accordingly, we examine the relationship of grandiose fantasizing to state self-esteem as well as to state positive and negative affect. Throughout the study, the strongest effects were for change in negative affect, followed by change in positive affect and change in state self-esteem, respectively. This suggests that in the short term, affect may be more closely connected to grandiose fantasizing than state self-esteem. Although this does not necessarily contradict prior literature, which largely focuses on self-esteem and bypasses any mention of affect, it does suggest that future work should consider the role of affect regulation in narcissism, even though self-esteem is central to theory. It is possible that the influence of grandiose fantasizing on self-esteem may be more salient in the long term, whereas its influence on affect may be stronger in the short term.

A critical question raised by this work is whether grandiose fantasizing is inherently maladaptive, as its clinical framing as a pathological trait would certainly suggest. The present finding that grandiose fantasizing leads to larger improvements in affect than does positive future thinking, regardless of narcissism levels, suggests that it may provide widespread, short-term affective benefits. However, the long-term effects are still unknown. It seems likely that the long-term impact may vary; indeed, Kohut (21) suggested that grandiose fantasy can contribute “to the success of the individual or to his downfall.” A central factor may be whether the fantasy serves a primary role of avoidance or motivation. For example, if someone frequently relies on a fantasy about being a professional athlete to avoid momentary feelings of negative affect or self-esteem, but fails to participate in athletics beyond the high school level, this may threaten their self-esteem over time and lead to feelings of shame or vulnerability [e.g., (54, 55)]. Further, they may be less likely to take action towards pursuing alternative careers. In contrast, if this fantasy motivates them to train hard each day and work towards their athletic goals, it may be extremely beneficial, even if they never reach a professional level.

Empirical work examining the functions of future thinking more broadly provides a framework for this perspective. Oettingen and Mayer (56) highlight the importance of differentiating between positive expectancy judgments and positive fantasies about the future. Whereas positive expectancy judgments are beliefs about the likelihood of future events grounded in past experiences, positive fantasies are images of future desired events that are pleasant to think about but not necessarily grounded in past experiences. In a series of experimental studies, positive expectancy judgements were associated with higher effort and successful performance, whereas positive fantasies were associated with lower effort and worse performance. Therefore, in considering whether a grandiose fantasy will be adaptive or maladaptive in the long-term, it may be critical to consider (1) whether the grandiose fantasy is grounded in past experience, and (2) whether the individual has taken action towards realizing the fantasy. We suspect that maladaptive grandiose fantasies held by individuals scoring high in narcissism will reflect a lack of grounding in reality and motivate little to no action, ultimately leading to long-term distress.

Although we cannot directly test whether the future thinking in the present study is reality-based or action motivating, independent external ratings of participant writing do provide a foundation for defining other features of grandiose fantasies. In comparison to positive future thinking, grandiose future thinking is characterized by low plausibility and high ambitiousness and agency. Additionally, grandiose future thinking tends to occur further in the future and generally has a more negative emotional tone than does positive future thinking. Grandiose future thinking was not characterized by more elaboration or detail, nor was it higher on distinctiveness, compared to positive future thinking. Future work should seek to replicate these findings and further explore how grandiose future thinking may be qualitatively different from more general positive thinking. It is also important to consider other forms of grandiose fantasy, such as present or past-focused fantasizing.

Methodological limitations of this study provide directions for future research. Our use of a forced choice paradigm, though effective in prompting grandiose future thinking in an experimental context, lacks ecological validity. Given that grandiose fantasies are highly individualized and may occur subconsciously, future work should aim to examine grandiose fantasizing in more naturalistic settings. For example, researchers could assess the relation of grandiose fantasizing to negative affect in daily life using ecological momentary assessment. Further, related research questions could be explored from a more qualitative perspective, such as interviewing participants to disentangle whether a positive future image is a motivating goal or maladaptive grandiose fantasy.

Similarly, it is important to recognize that not all responses to grandiose writing prompts in the current study reflect a grandiose fantasy. For example, a participant writing about a time they would feel powerful wrote about lifting weights at the gym the next day, a decidedly non-grandiose future image. Regardless, the consistent group differences between the secondary ratings (e.g., ambitiousness, plausibility) of the grandiose vs. positive future writing suggest that overall, the word choice manipulation led to systematically different types of future thinking. Future work should aim to continue developing more specific techniques to evoke grandiose fantasies in an experimental setting.

Our current sample size limits the ability to test possible mechanisms underlying the central findings. Future work should explore *why* grandiose fantasizing is a more effective affect regulator for those scoring higher vs. lower in narcissism. For example, it is likely that grandiose fantasizing is a better affect regulator for those high in narcissism if they imagine the event with more clarity, are more likely to believe it will occur, or perhaps envision it as occurring sooner, than those low in narcissism. Differences in the phenomenology of the grandiose fantasy (e.g., detail, visual perspective), frequency of engaging in the fantasy, or perceived plausibility of the fantasy would be valuable to explore as potential moderators. Further, given the limited sample size, all findings should be replicated to increase confidence in their validity. As the present sample was two-thirds White and entirely based in the United States, a more racially and geographically diverse sample would also strengthen the generalizability of future replications.

To our knowledge, this is the first study to demonstrate that that people scoring high in narcissism are more likely to *choose* to engage in grandiose fantasizing to make themselves feel better. We also believe that this is the first study to demonstrate that grandiose fantasizing is a more efficacious affect regulator for people scoring high in narcissism than people scoring low in narcissism. This work provides empirical support for an oft-cited theory that grandiose fantasizing is regulatory and should increase confidence in the idea that grandiose fantasizing is not just a general tendency in pathological narcissism, but rather serves a functional (and potentially adaptive) role. It also highlights the specificity of this function for people scoring higher in narcissism and suggests that affect may be a variable that warrants increased consideration in future research. Although we provide evidence supporting the short-term benefits of grandiose fantasizing, future work would do well to also consider its longer-term consequences.

## Data availability statement

The raw data supporting the conclusions of this article will be made available by the authors, without undue reservation.

## Ethics statement

The studies involving humans were approved by Harvard University-Area Institutional Review Board (HUA-IRB). The studies were conducted in accordance with the local legislation and institutional requirements. The participants provided their written informed consent to participate in this study.

## Author contributions

EF: Conceptualization, Formal analysis, Funding acquisition, Investigation, Methodology, Writing – original draft. JH: Conceptualization, Supervision, Writing – review & editing, Methodology.

## Footnotes

^2^The grandiose words from the NGS are reliable and valid predictors of narcissistic grandiosity and strongly differentiate narcissistic grandiosity from self-esteem (44).
