# Supplementary material for: Functional fantasies: the regulatory role of grandiose fantasizing in pathological narcissism
Source: Front Psychiatry. 2023 Oct 18;14:1274545. doi: 10.3389/fpsyt.2023.1274545 (PMC10618345; doi:10.3389/fpsyt.2023.1274545)
Supplement: Supplementary file 1 [file Table_1.DOCX]

**Table S1**

*Demographics Table*

|  | Primary Sample  (*N* = 189) | Secondary Sample  (*N* = 128) |
| --- | --- | --- |
|  | n (%) | n (%) |
| *Gender* |  |  |
| Cisgender Female | 94 (49.74) | 62 (48.44) |
| Cisgender Male | 89 (47.09) | 63 (49.22) |
| Non-binary/genderqueer | 1 (<1.00) | 3 (2.34) |
| Multiple gender identities | 2 (1.06) | 0 (0.00) |
| Transgender Female | 3 (1.59) | 0 (0.00) |
| Transgender Male | 0 (0.00) | 0 (0.00) |
| *Race* |  |  |
| American Indian or Alaskan Native | 2 (1.06) | 0 (0.00) |
| Asian | 14 (7.41) | 10 (7.81) |
| Black or African American | 16 (8.47) | 15 (11.72) |
| Hispanic, Latino or Spanish | 11 (5.82) | 10 (7.81) |
| Middle Eastern or North African | 3 (1.59) | 0 (0.00) |
| More Than One Race | 16 (8.47) | 14 (10.94) |
| Native Hawaiian/Pacific Islander | 0 (0.00) | 1 (<1.00) |
| Other | 0 (0.00) | 1 (<1.00) |
| Prefer not to answer | 1 (<1.00) | 0 (0.00) |
| White | 126 (66.66) | 77 (60.16) |
| *Sexual Orientation* |  |  |
| Bisexual | 22 (11.64) | 20 (15.62) |
| Gay/Lesbian/Homosexual | 7 (3.70) | 10 (7.81) |
| Heterosexual/Straight | 151(79.90) | 92 (71.88) |
| Unsure | 3 (1.59) | 1 (<1.00) |
| Pansexual | 6 (3.17) | 5 (3.91) |
| *Employment Status* |  |  |
| Employed Full Time | 95 (50.26) | 61 (47.66) |
| Employed Part Time | 26 (13.76) | 16 (12.50) |
| Homemaker | 7 (3.70) | 7 (5.47) |
| Self-employed | 12 (6.35) | 8 (6.25) |
| Student | 17 (8.99) | 15 (11.72) |
| Unemployed; currently looking for work | 22 (11.64) | 20 (15.63) |
| Unemployed; not currently looking for word | 10 (5.29) | 1 (<1.00) |
|  | *M* (*SD*) | *M* (*SD*) |
| *Age (years)* | 29.48 (5.73) | 28.89 (5.91) |
